# Supplementary material for: Impact of In Vitro Digestion on the Digestibility, Amino Acid Release, and Antioxidant Activity of Amaranth (Amaranthus cruentus L.) and Cañihua (Chenopodium pallidicaule Aellen) Proteins in Caco-2 and HepG2 Cells
Source: Antioxidants (Basel). 2023 Dec 5;12(12):2075. doi: 10.3390/antiox12122075 (PMC10740650; doi:10.3390/antiox12122075)
Supplement: Supplementary file 1 [file antioxidants-12-02075-s001.zip › antioxidants-2711459-supplementary.pdf]

Supplementary data

Table S1. Two-way ANOVA analysis values of percentage of digestibility of *Amaranthus cruenthus* and *Chenopodium pallidicaule* proteins, considering the effect of the Food matrix and digestion time.

	df	*F*
Food Matrix	1	186.31*
Digestion Time	8	409.23**
Food matrix x Digestion Time	8	13.84*
Error	36	

Values with asterisks represent $*p < 0.01$, $**p < 0.001$

Table S2. Two-way ANOVA analysis values of effect of *in vitro* gastrointestinal digestion on amino acid profile (mg g^{-1} protein) of *Amaranthus cruenthus* (A) and *Chenopodium pallidicaule* (B) proteins considering the effect of the Food Matrix and Digestion phase.

**A**	df	*F*
Amino acids	16	2687.4**
Digestion phase	1	108209.4**
Amino acids x Digestion phase	16	2071.3**
Error	68	

**B**	df	*F*
Amino acids	16	7396.3**
Digestion phase	1	290259.1**
Amino acids x	16	6168.9**
Error	68	

Values with asterisks represent $**p < 0.001$

Table S3. Two-way ANOVA analysis values of antioxidant activity by ORAC, ABTS and DPPH assays, considering the effect of the Food matrix and digestion phase.

		ORAC	ABTS	DPPH
	df	*F*	*F*	*F*
Food matrix	2	530.57**	6.42*	171.9**
Digestion phase	1	75.2**	3204.2**	131.8**
Food matrix x Digested	2	22.44**	4.5*	32.9**
Error	12			

Values with asterisks represent *$p < 0.01$, **$p < 0.001$

Table S4. Source of variation of the three-way ANOVA model for cellular antioxidant activity in HepG2 (A) and Caco-2 cells (B) comparing the three factors: Food matrix, Sample and Concentration, and their respective interactions.

**A** Source of variation	df	*F*	*p*	Post hoc
Food matrix	1	7.214	0.01	A = C
Concentration	1	734.422	0.001	0.750 > 0.250
Sample	5	324.828	0.001	U, G<5, G>5, I<5, I>5 > C
Food Matrix x Concentration	1	2.850	0.097	
Food Matriz x Sample	5	4.983	0.001	
Concentration x Sample	5	40.720	0.0	NS
Food matrix x Concentration x Sample	5	0.656	0.656	
Error	48			

**B** Source of variation	df	*F*	*p*	Post hoc
Food Matrix	1	0.030	0.862	A = C
Sample	5	445.609	0.001	U, G<5, G>5, I<5, I>5 > C
Concentration	1	3948.590	0.001	0.750 > 0.250
Food Matrix x Sample	5	3.876	0.004	
Food Matrix x Concentration	1	0.024	0.877	
Sample x Concentration	5	183.094	0.0	NS
Food Matrix x Sample x Concentration	5	3.906	0.004	
Error	48			

NS= not significant
